# Oral motor deficits in speech-impaired children with autism

**DOI:** 10.3389/fnint.2013.00047

**Published:** 2013-07-01

**Authors:** Matthew K. Belmonte, Tanushree Saxena-Chandhok, Ruth Cherian, Reema Muneer, Lisa George, Prathibha Karanth

**Affiliations:** ^1^The Groden Center, ProvidenceRI, USA; ^2^Division of Psychology, Nottingham Trent UniversityNottingham, UK; ^3^Center for the Study of Human Development, Brown University, ProvidenceRI, USA; ^4^The Com DEALL TrustBangalore, India

**Keywords:** autism, non-verbal, oral motor, speech, language, dyspraxia

## Abstract

Absence of communicative speech in autism has been presumed to reflect a fundamental deficit in the use of language, but at least in a subpopulation may instead stem from motor and oral motor issues. Clinical reports of disparity between receptive vs. expressive speech/language abilities reinforce this hypothesis. Our early-intervention clinic develops skills prerequisite to learning and communication, including sitting, attending, and pointing or reference, in children below 6 years of age. In a cohort of 31 children, gross and fine motor skills and activities of daily living as well as receptive and expressive speech were assessed at intake and after 6 and 10 months of intervention. Oral motor skills were evaluated separately within the first 5 months of the child's enrolment in the intervention programme and again at 10 months of intervention. Assessment used a clinician-rated structured report, normed against samples of 360 (for motor and speech skills) and 90 (for oral motor skills) typically developing children matched for age, cultural environment and socio-economic status. In the full sample, oral and other motor skills correlated with receptive and expressive language both in terms of pre-intervention measures and in terms of learning rates during the intervention. A motor-impaired group comprising a third of the sample was discriminated by an uneven profile of skills with oral motor and expressive language deficits out of proportion to the receptive language deficit. This group learnt language more slowly, and ended intervention lagging in oral motor skills. In individuals incapable of the degree of motor sequencing and timing necessary for speech movements, receptive language may outstrip expressive speech. Our data suggest that autistic motor difficulties could range from more basic skills such as pointing to more refined skills such as articulation, and need to be assessed and addressed across this entire range in each individual.

## Introduction

Deficits in communication have long been recognised as an essential characteristic of autism, earning a place in the triad of diagnostic signs. Autism is, however, a developmental disorder not only nosologically but also ætiologically, and therefore the deficits that are most obvious, most diagnostic, and most debilitating might not necessarily be the most ætiologically primary. Viewing autism as a developmental disorder, then, compels one to seek beyond the developmental endpoints on which diagnosis is based, to identify root causes. Evidence and interpretation as to the cause of the communication deficit have ranged from a lack of social motivation or social reward (Chevallier et al., 2012), with the social cognitive capacity to develop communication presumably being intact, to specific issues in social cognition including pragmatic applications of communicative skills (Tesink et al., 2009) or theory-of-mind and perspective-taking (Frith, 1997). Debates on autism's origins, therefore, often end up framed in terms of differences between social motivational and social cognitive theories. Of course, as autism is a behaviourally diagnosed syndrome with a great degree of heterogeneity in presentation, it's likely to admit many biological causes, with different combinations of these biological causal mechanisms converging into one and the same set of diagnostic behavioural traits, and diverging into variation within the behaviourally defined phenotype (Belmonte et al., 2004). These putative causal mechanisms of social motivation and social cognition must not, therefore, be approached as exclusive of each other—or of other, even more fundamental causal mechanisms.

In both these sets of accounts, the cognitive and the motivational, the developmental endpoint combines disruptions of social communication and social reward, the only distinction being which one of these symptoms arises first and incurs the other. Seldom has the autistic disruption of social communication been conceptualised as a consequence of difficulties in acquiring and producing speech and language. Evidence to the contrary, that is, acknowledgement that at least in a subpopulation of children with autism communicative deficits may instead stem from more basic motor and oral motor issues, is now emerging. Qualitative and quantitative assessments of gross, fine, and oral motor functions in children with autism as compared to their neurotypical peers have recorded significant differences, suggesting that motor deficits could underlie some of autism's communicative and social symptoms [see Leary and Hill (1996) for a review]. A case is therefore increasingly made for screening children with autism for neuro-motor deficits and for addressing these in intervention where appropriate (Noterdaeme et al., 2002).

Amongst the motor skills, oral motor skills in particular are closely linked with speech production, fluency and clarity. Here too recent research is documenting the association between early oral motor skills and later speech fluency. Amato and Slavin (1998) noted the link between oral motor movements involving the tongue and lips and speech fluency in children with autism. Similar measures are in fact reported to be sufficiently robust as to distinguish autistic children from typically developing children, and also to distinguish between autistic children with eventually varying degrees of fluency (Gernsbacher et al., 2007). In children whose non-verbal cognitive skills are relatively intact, vocal, and other motor imitation skills at early ages—even more so than early joint attention—predict language skills at the age of 5 years (Thurm et al., 2007).

Intensive early intervention (EI) for children with autism has been shown to make a clinically significant difference for many children in multiple areas including language. The Communication DEALL EI (Karanth, 2010; Karanth et al., 2010) programme provides intensive intervention for young children (0–6 years) with autism spectrum disorders via an interdisciplinary team comprising a speech language therapist, an occupational therapist and a developmental educator/psychologist. Developmental skills are assessed and strengthened in eight domains including gross motor (GM), fine motor (FM) and activities of daily living (ADL), receptive language (RL), expressive language (EL), cognitive (C), social (S) and emotional (E) skills. Additional skills including pre-requisite learning skills (PLS), oral motor skills (OM), sensory issues (SI), and pragmatic skills are also assessed and targeted at different stages of the programme. Assessments are conducted at three intervals for each child—immediately prior to intervention (initial assessment), 6th month of intervention (mid assessment) and the 10th month (final assessment).

Our early-intervention programme develops skills prerequisite to learning and communication, including eye contact, joint attention, sitting tolerance, and compliance along with pointing or reference. Once the child shows improvement in these prerequisite learning skills, intervention tailored to the individual student's profile is provided across all domains. Over several years of clinical experience we have observed anecdotally that toddlers and young children with motor difficulties including oral motor difficulties seem more likely to remain non-verbal or to have persistent difficulties in expressive speech and language development. The increasing disparity between receptive and expressive speech and language abilities in this subgroup of children reinforces the hypothesis that, in these cases, expressive or speech deficits may be secondary to oral motor deficits. This study was undertaken to ascertain quantitatively the existence, nature, and proportion of such a subgroup amongst children diagnosed with autism within our clinical population. From a clinical viewpoint, such knowledge is a prerequisite to developing an intervention that targets this subpopulation's underlying issues early and specifically. From a pure research viewpoint, this closer characterization may help to disentangle the heterogeneity in autism's detailed phenotypes and causes.

In selecting assessments for any such clinical study a balance must be struck between the clinical measures most germane and appropriate to the clinical population and its therapeutic needs, on the one hand, and the research measures standardised and normed against typically and atypically developing populations worldwide. We have chosen to apply two indigenously developed clinical measures germane to the Indian therapeutic setting. Although cross-validation against measures developed in other cultures remains to be conducted, these measures have been normed and validated within India, have been reported in the peer-reviewed literature and codified as clinical manuals, are sensitive to the Indian population, are culturally appropriate, and emphasise clinical utility.

## Materials and methods

### Subjects

Data collection took place as part of a cross-cultural comparative study of autism spectrum conditions approved by the Institutional Review Board of the Groden Center, and informed consent was obtained from each parent for research use of their children's clinical data. Case files of all children enrolled from 2009 to 2011 were reviewed, and diagnoses of autism confirmed by reference to ICD-10 criteria (World Health Organization, 1993). Cases for whom ICD-10 diagnosis of autism was in any doubt were excluded, yielding a study population of 31 children (6 females, 25 males, 4:1 male:female ratio) of middle to high socioeconomic status. Ages at enrolment ranged from 22 to 65 months, with a mean of 41 months and a standard deviation of 11 months.

Subjects attended at least one year of daily intervention with consistent monitoring at an early intervention centre and were assessed thrice (pre/mid/post-intervention) within the year. Along with the aforementioned prerequisite learning skills, the beginning of the early intervention programme addresses issues of feeding and toileting, if present. Subsequently, intensive inputs in the domains of communication, motor and cognitive, social and emotional skills are provided daily throughout the year (Karanth, 2010). It has been our clinical experience that at this stage, 2–3 months into the programme, receptive language skills begin to improve. At the same time we see a differential effect in terms of expressive language skills: Whilst in one subgroup, gains in expressive language appear commensurate with those in receptive language, in another subgroup expressive language skills are far lower. Children in this latter, expressive-impaired group are provided with more directed oral motor intervention, comprising activities related to management of oral sensory issues, improvement of tone, massages, exercises and oral motor games [see Aluri (2005), for details]. All oral motor exercises are done by the same team 2–3 times per week, with follow-up by parents.

### Tools

Two assessment instruments developed in India and normed for Indian populations were applied:

#### The Com DEALL developmental checklist (CDDC)

The CDDC (Karanth, 2007) is a criterion referenced checklist to assess developmental skills in 8 domains—namely, gross and fine motor skills, activities of daily living, receptive and expressive language skills, and cognitive, social and emotional skills—at 6 month intervals, from 0 to 6 years of age. Questions in each domain are further subdivided in 12 age sub-groups from 0–6 months to 66–72 months. The checklist includes 36 items in each of the 8 domains assessed, for a total of 288 items. The CDDC has been field tested on urban Indian children from middle class backgrounds, has a high inter-rater reliability, and can be used as a screening measure for identification of developmental delays in specific domains (Karanth et al., 2010). The CDDC thus carries face and content validity, and shows convergent validity with independent Childhood Autism Rating Scale diagnoses (Karanth et al., 2010).

#### The Com DEALL Oro motor assessment

Children with speech language acquisition delays and disorders often have difficulties in oral motor skills. This checklist (Archana, 2008) is a standardised tool for assessing oral motor skills of children within the range of 1–4 years. It has been designed to identify clinically children who have oral motor problems, by providing developmental norms, and to inform the development of goals for intervention. It assesses 4 domains—jaw, tongue, and lip movements and speech. The 30 items cover an observation and assessment of the articulators in terms of posture (open mouth posture/extended tongue), movement (transitions from one movement to the other/raising of the tongue), function (biting/sucking), and speech production at the level of combinations of vowels and consonants in syllables, words, and phrases of varying length and complexity. All items are rated on a three-point scale, from absent, to only present spontaneously, to consistently present (on demand). For further details see (Archana, 2008). The norms are based on field testing of 90 urban Indian children.

### Procedure

Data collected from each case file comprised age at enrolment and raw scores along the three time points (pre-, mid-, post-intervention) for the five domains of interest: gross motor, fine motor, receptive language, expressive language, and oral motor. All daily interventions and periodic assessments were carried out by the team assigned to the group of children. This team was composed of the same clinical staff throughout all time points of measurement. The team consists of an occupational therapist, a speech language pathologist and a developmental educator/psychologist. The oral motor assessment was conducted jointly by the occupational therapist and the speech language pathologist.

Raw scores at each time point were converted to percentages by dividing by the total number of applicable items. Non-compliance in a few subjects prevented acquisition of oral motor scores from one or another time point; the mid-intervention score was unavailable from 6 subjects, and the pre-intervention score was unavailable from 3 subjects. Although the children's specific reasons for non-compliance with the oral motor tests cannot be proven, it was the impression of the clinical team that these cases of non-compliance arose because of sensory sensitivities triggered by the assessment procedures. The mouth and lips being a zone rich in tactile input, this oral motor assessment is *a priori* the most likely of our procedures to trigger tactile aversion in sensitive individuals. In contrast, had non-compliance been a consequence of receptive language difficulties it would have been equally likely to arise in the other, non-oral-motor assessments rather than arising specifically in the oral motor context. In these cases in which one of the three observations was missing because of non-compliance, slopes of the intervention scores over time were estimated from the two other time points. Scores for all measures other than these oral motor assays were available at all time points for all subjects.

### Statistical analysis

**Figure 1:**
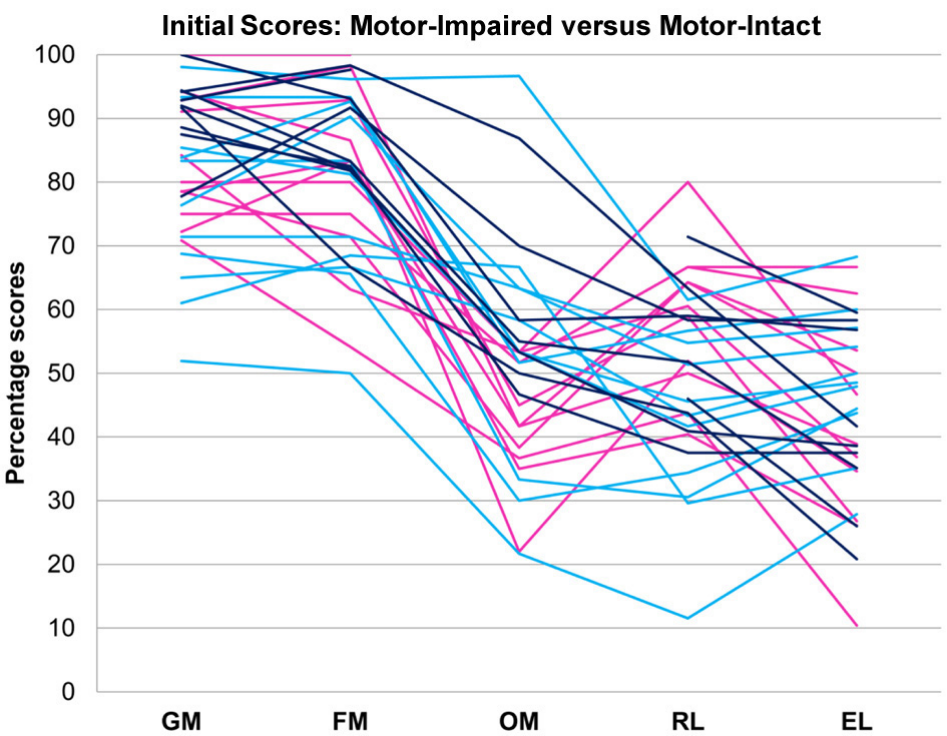
**Motor-impaired (**
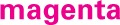
**) and motor-intact (blue, with receptive-impaired subgroup in** 
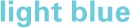
 **and receptive-intact subgroup in** 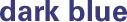
**) groups**. The discriminant between motor-impaired and motor-intact groups −0.28·OM − 0.15·GM + 0.55·RL − 0.20·EL loaded heavily on the receptive-expressive difference and on oral motor skills, and also slightly on gross motor skills. GM, gross motor; FM, fine motor; OM, oral motor; RL, receptive language; EL, expressive language.

On the basis of the therapeutic team's clinical impression, the 31-subject sample was classified into a motor-impaired group (11 subjects) in whom expressive language difficulty seemed to occur along with oral motor impairments out of proportion to impairments in other domains, and a motor-intact group (20 subjects) in whom no such uneven profile existed (Figure 1). The two groups did not differ in age [motor-impaired 37.45 ± 14.36 months at enrolment, range 22–65 months, and motor-intact 43.20 ± 8.55 months at enrolment, range 29–58 months, *t*_(29)_ = 1.40, *p* = 0.1711]. As an exploratory characterization, the motor-intact group was further subdivided into a receptive-impaired subgroup with receptive language deficit out of proportion to expressive language impairments, and a receptive-intact subgroup in whom receptive and expressive language skills were on par.

Slopes for all measures as functions of time were computed from the three (or in cases of missing oral motor data, two) time points, treating the time intervals between the first and second and the second and third observations as equal.

A linear discriminant function was constructed (SAS PROC DISCRIM, POOL=yes CROSSVALIDATE) to distinguish the motor-impaired and motor-intact groups. This procedure was attempted with three sets of inputs: once with pre-intervention values and slopes of all variables, once with slopes only, and once with pre-intervention values only. The pre-intervention values, without slopes, yielded the most accurate discrimination as assayed by leave-one-out cross-validation. Single measures then were deleted one by one from the linear discriminant input, to determine whether they were essential to discrimination. This procedure yielded a discriminant function with 100% selectivity and specificity, loading negatively on gross and oral motor skills and expressive language, and positively on receptive language. This discriminant function and its slope over time were added to the data set as derived measures. Also added as derived measures were the difference between receptive and expressive language scores, which discriminated the receptive-impaired subgroup from the receptive-intact subgroup within the motor-intact group with 100% selectivity and specificity, and the slope of this receptive-expressive difference.

Pre-intervention values and slopes of all observed and derived measures were correlated against each other. As the study was motivated by the hypothesis that expressive impairment out of proportion to receptive impairment may be secondary to oral motor impairment, correlations between oral motor and expressive skills were evaluated as planned comparisons, the other correlations as exploratory.

Outcome differences between groups were assayed via analyses of variance for each observed measure. Dependent variables were the post-intervention values of all observed measures, and the differences between pre-intervention and post-intervention values. In the three cases in which the pre-intervention oral motor score was unavailable, the mid-intervention score was used in computing this difference. Again oral motor and expressive language scores were treated as planned comparisons between motor-impaired and motor-intact groups. In addition, receptive and expressive language scores were treated as planned comparisons between the clinically classified receptive-impaired and receptive-intact subgroups of the motor-intact group. Other measures were treated as exploratory.

## Results

Pre-intervention score profiles for the motor-impaired and motor-intact groups are illustrated in Figure 1, which contains one series of line segments for each individual subject, within each of the groups, linking that individual's gross motor, fine motor, oral motor, receptive language and expressive language skills. Reading the line segments from left to right highlights scores that are out of proportion to the individual subject's overall level of functioning: Note the dips in oral motor (“OM”) and expressive language (“EL”) scores for members of the motor-impaired group as contrasted with members of the motor-intact group. Slopes did not contribute to the accuracy of the linear discriminant between motor-impaired and motor-intact groups, nor did fine motor scores. The final discriminant, based entirely on pre-intervention measures, reliably separated (100% sensitivity and specificity with leave-one-out cross-validation) the motor-impaired and motor-intact groups, loading negatively on oral motor skills (coefficient −0.28) and also slightly negatively on gross motor skills (−0.15), and heavily positively on the receptive-expressive language difference (+0.55 and −0.20, respectively). The gross motor score made for a slightly more accurate discriminant than the fine motor, and addition of the fine motor measure, which was highly correlated with gross motor, did not improve discrimination. The distribution of this discriminant function was bimodal (Table 1), with normal modes corresponding to the motor-intact and motor-impaired groups. The learning rate (slope) for receptive language was highly correlated with the motor-intact/impaired discriminant function, with the motor-impaired group learning much more slowly than the others (Table 2; see also Figure 3).

**Table 1:**
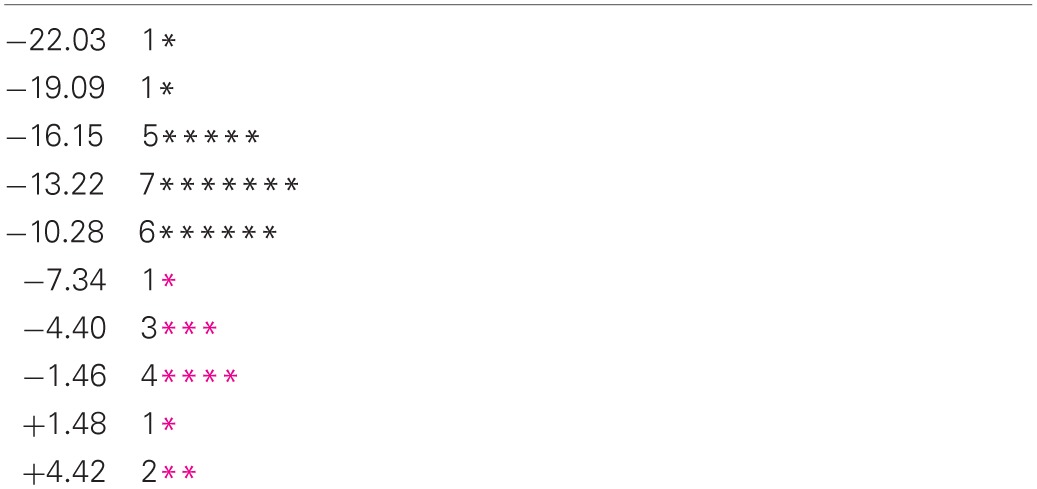
**Histogram of values of the discriminant function −0.28·OM − 0.15·GM + 0.55·RL − 0.20·EL for members of the motor-impaired (**
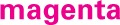
**) and motor-intact (black) groups**.

**Table 2:**
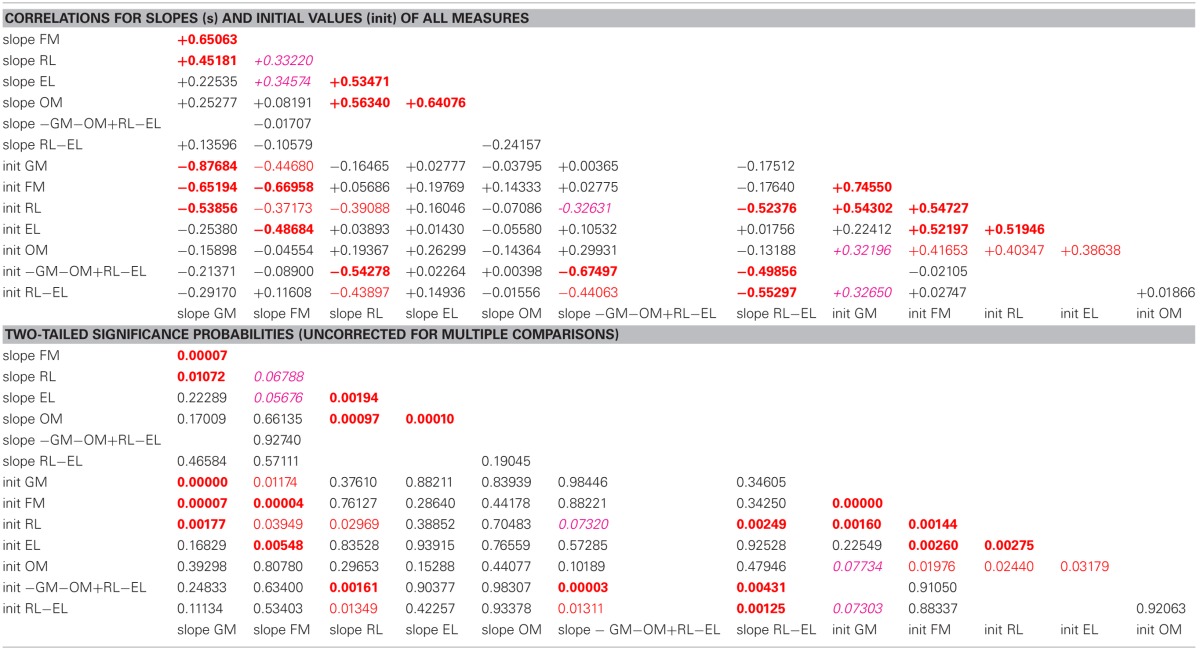
**Correlation coefficients and associated two-tailed probabilities for slopes (s) and initial values (init) of all observed and derived measures, uncorrected for multiple comparisons**.

*Pairs collinear by definition have been deleted. Correlations statistically significant after correction for multiple comparisons are indicated in* 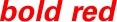, *exploratory correlations not significant after correction for multiple comparisons are in* 
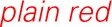
, *and suggestive trends in* 
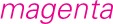
. *GM, gross motor; FM, fine motor; OM, oral motor; RL, receptive language; EL, expressive language; init, initial value; slope, slope across time points. “−GM−OM+RL−EL” denotes the discriminant function −0.28·OM − 0.15·GM + 0.55·RL − 0.20·EL.*

In the pre-intervention scores of the sample as a whole, gross and fine motor skills and receptive language were highly correlated with each other, and expressive language was correlated with fine (but not gross) motor skills. Oral motor skills were correlated, less strongly, with fine motor and receptive and expressive language. The learning rates (slopes) for expressive and receptive language were highly correlated with the learning rate for oral motor skills.

The motor-intact group were further characterised into two overlapping subgroups by disparity in receptive and expressive language scores. The distribution of this receptive-expressive score difference was again bimodal (Table 3), though the two modes were not cleanly separated, with the lesser mode comprising mostly the receptive-impaired subgroup and the greater mode including the receptive-intact subgroup along with the motor-impaired group.

**Table 3:**
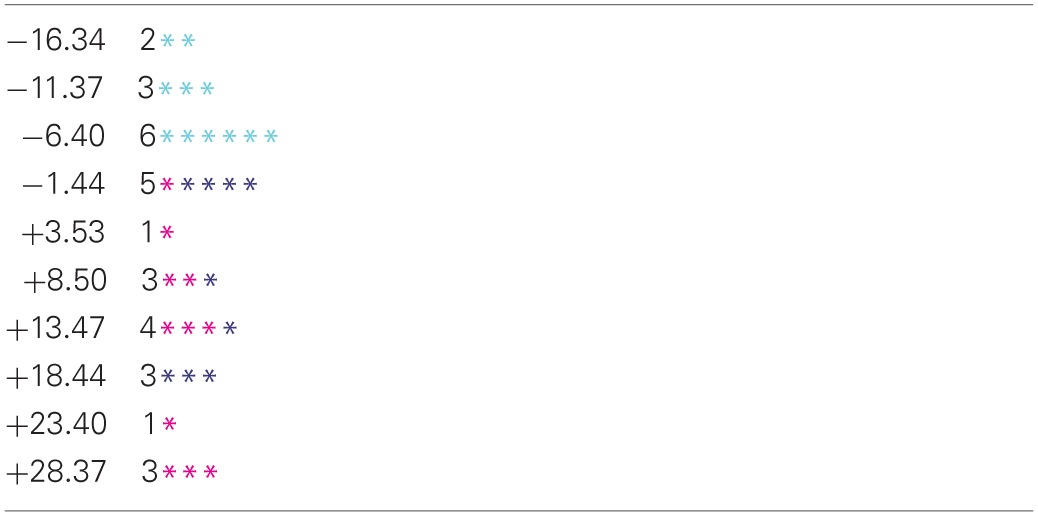
**Histogram of values of the receptive-expressive language difference (RL-EL) for the motor-intact receptive-impaired (**
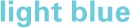
**) and motor-intact receptive-intact (**
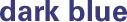
**) subgroups, and the motor-impaired (**
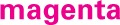
**) group**.

**Figure 2:**
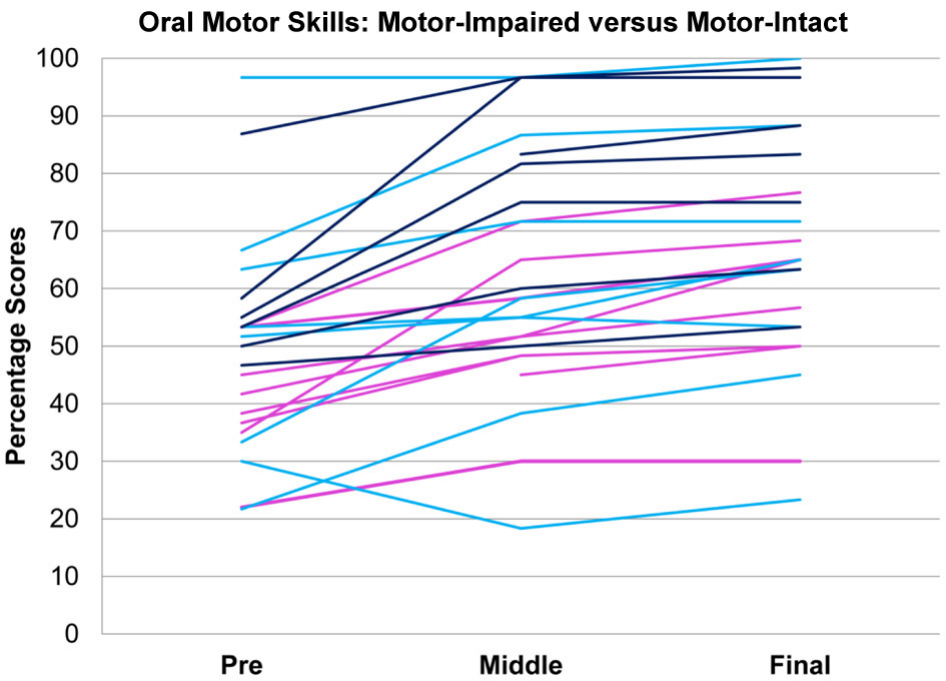
**Oral motor scores over time in motor-impaired (**
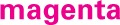
**) and motor-intact (**
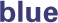
**) groups**. Modulo a great deal of heterogeneity, the motor-impaired group on the whole began with lesser scores than the motor-intact group, by definition, but also ended with lesser scores. (For consistency with the other figures, the motor-intact group is color-coded separately as 
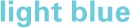
 for the receptive-impaired subgroup and 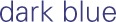 for the receptive-intact).

**Figure 3:**
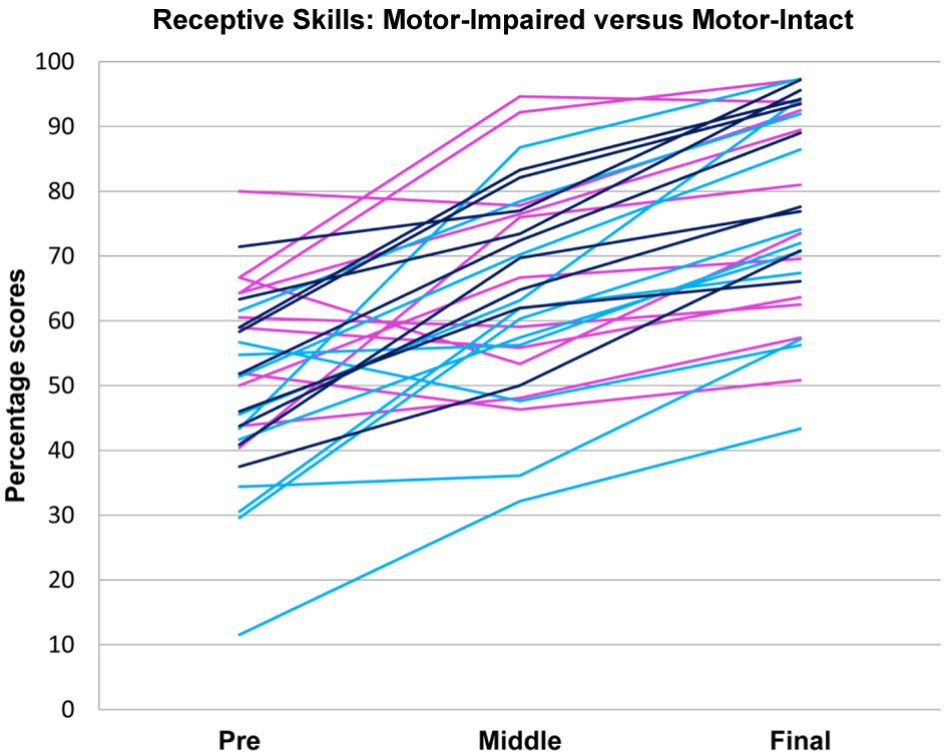
**Receptive language scores over time in motor-impaired (**
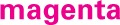
**) and motor-intact (**
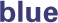
**) groups**. The motor-impaired group improved less than the motor-intact group; note the group difference in line slopes. (For consistency with the other figures, the motor-intact group is color-coded separately as 
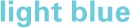
 for the receptive-impaired subgroup and 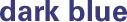 for the receptive-intact.)

**Figure 4:**
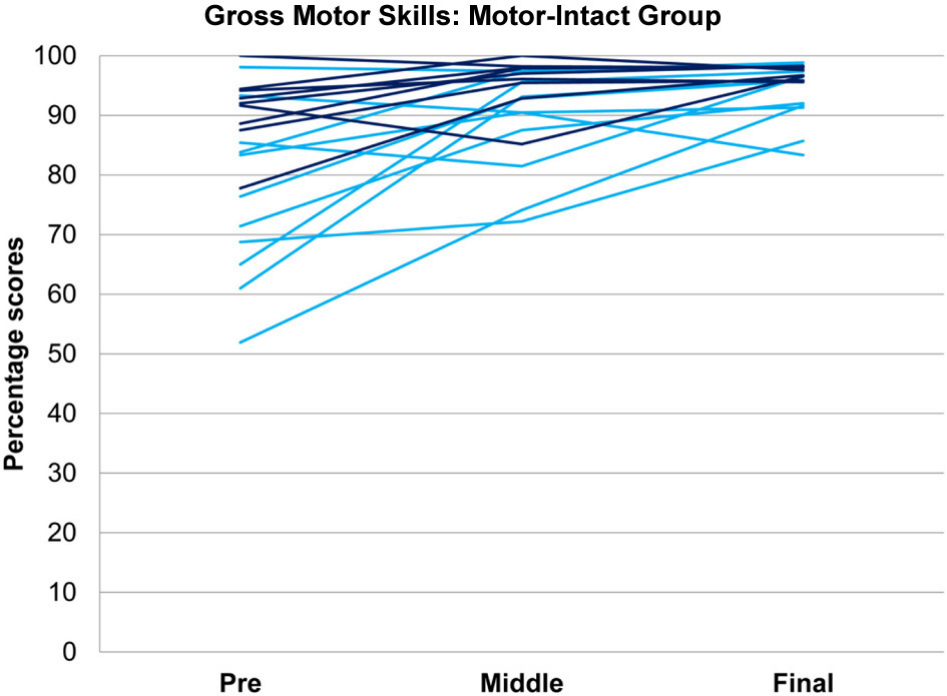
**Gross motor scores over time in the receptive-impaired (**
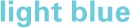
**) and receptive-intact (**
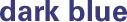
**) subgroups of the motor-intact group**. The receptive-impaired group ended the intervention with lesser gross motor scores, and this difference was driven by many individuals who began with more severe gross motor deficits and, though they improved at rates similar to those of the receptive-intact subgroup, did not yet approach ceiling by the end of the intervention.

In tests of group differences in outcome, the motor-impaired was distinguished from the motor-intact group by a lesser post-intervention oral motor score [motor-impaired 59.85 ± 16.62, motor-intact 75.50 ± 20.66, *F*_(1, 29)_ = 10.85, *p* = 0.0026, Figure 2] and also by a lesser pre-post difference in receptive language score [motor-impaired 16.72 ± 13.51, motor-intact 31.94 ± 11.63, *F*_(1, 29)_ = 4.64, *p* = 0.0398, Figure 3]. Within the motor-intact group, the receptive-impaired was marginally distinguished from the receptive-intact subgroup by a lesser post-intervention gross motor score [receptive-impaired 76.22 ± 14.11, receptive-intact 91.00 ± 6.13, *F*_(1, 18)_ = 8.49, *p* = 0.0093, Figure 4], and this difference seemed driven by many receptive-impaired individuals who began the intervention with more severe gross motor impairments and, though they progressed at rates similar to those of the receptive-intact subgroup, had not yet caught up by intervention's end. There also was a trend towards a greater pre-post difference in oral motor score [receptive-impaired 8.57 ± 7.45, receptive-intact 3.10 ± 2.96, *F*_(1, 18)_ = 4.26, *p* = 0.0538].

## Discussion

Results confirm the clinical impression that in a third of this sample, 11 of the 31 consecutively enrolled subjects with unequivocal ICD-10 diagnoses of autism, a disparity between receptive language skill and expressive speech impairment is associated with oral and other motor impairments. Motor-intact and motor-impaired groups were distinguished by a discriminant with positive loading on receptive-expressive language disparity and oral motor skills, and also somewhat on gross motor skills which were in turn highly correlated with fine motor skills. This function gives quantitative basis to the clinically observed difference between the two groups, exactly separating them into two distinct modes.

Following the period of intervention the motor-impaired group did not achieve as proficient post-intervention oral motor function, and across the entire sample the learning rates for both receptive and expressive language were highly correlated with the learning rate for oral motor skills. Even before intervention began, receptive language was correlated with gross and fine motor skills, and both receptive and expressive language were correlated with fine and oral motor skills.

Our results reinforce the notion that many people with autism experience substantial motor difficulties including deficits in gross motor, fine motor, and oral motor skills, despite the subtle presentation of these motor deficits in the context of much more obvious social cognitive symptoms, particularly at young ages. Whilst sensory issues in children on the autism spectrum have received considerable attention of late, the motor issues have not and need to be assessed in all children with autism spectrum conditions whether they appear to have motor difficulties or not. It is noteworthy that similar motor issues were neglected initially in children with a diagnosis of specific language impairment (SLI) only to be identified and documented subsequently (Hill, 2001; Marton, 2009; Rechetnikov and Maitra, 2009; Zelaznik and Goffman, 2010); in one recent study fully one third of children with SLI satisfied criteria for an additional diagnosis of Developmental Coordination Disorder (Flapper and Schoemaker, 2013). Behavioural study of twin pairs suggests a partly genetic basis for covariation of clinical communicative impairment and motor (finger-tapping) impairment (Bishop, 2002). In a large (62,944 individuals) sample of typically developing children, too, motor skills at age 1.5 years correlate with communicative skills, and predict communicative skills at age 3 (Wang et al., 2013). Speech and language acquisition in particular, seem closely linked to mastery of oral motor skills in a subgroup of children with autism. Within this subgroup, lack of expressive language skills or speech in particular, in the presence of relatively better receptive language skills, is highly correlated with poor oral motor skills.

The overall progress that children with autism make appears related to their progress in mastering and overcoming their motor issues. Our results indicate that not only do the motor deficits correlate highly with *level* of speech-language acquisition prior to intervention, but in addition the severity of the motor deficits could influence the overall rate of learning, particularly the learning of expressive language as the learning rates for expressive and receptive language were highly correlated with the learning *rate* for oral motor skills. Oral motor issues when present could pose a considerable challenge to the acquisition of speech, as the motor-impaired group was distinguished from the motor-intact group by a lesser post-intervention oral motor score. Moreover, oral motor skills in this sample vary somewhat *independently* of gross and fine motor skills, being only weakly correlated in initial level, and not at all significantly correlated in rates of development. These outcomes and characteristics highlight the need not only for individual assessment of the gross, fine, and oral motor skills in children with autism spectrum conditions but even more importantly the need for focused, individualised and child-centred intervention in all of these areas, including oral motor skills.

This small clinical study is of course not without its limitations. As this study did not involve a clinical control group, we are unable to evaluate how the therapy itself might affect the results. It was the pre-intervention motor and language scores that most effectively discriminated the motor-impaired from the motor-intact group. The question remains open, then, as to whether the same population with no intervention at all, or with an intervention not targeting oral motor skills, might spontaneously close the gap in expressive language between these motor-impaired and motor-intact groups. This study aimed not at evaluating the therapy itself—which already has been the subject of past reports—but rather at discriminating and characterizing this motor subgroup. The discriminant based on pre-intervention scores does speak to this objective.

In addition, though the measures of motor function used in this study have been evaluated and normed within India, they have not yet been cross-validated against worldwide standards such as the Mullen Scales of Early Learning or the Vineland Adaptive Behavior Scales (VABS). One of the obstacles to such cross-validation is the cost of the scales themselves which is often prohibitive for non-governmental organizations operating in developing countries (Durkin, 2013). Norming of the Mullen and/or the VABS against the CDDC and the Com DEALL Oro Motor Assessment would be a next logical step, as would a controlled study in which individuals would be randomised to distinct intervention groups so as to assay interactions between motor-impaired or motor-intact starting point, intervention, and outcome.

Correlations between speech and motor skills can arise from motor impairments *per se*, or from disconnection between motor execution and executive planning and sequencing (Hill, 2004) and/or affective motivation (Greenspan, 2001). It remains unclear from the results reported here whether the issue within the motor-impaired group might be one of oral motor execution, or of cognitive and/or affective control: that is, might autistic people with the ability to vocalise be unable to connect that ability to willed communication? This question of course relates to the debate mentioned in our introduction, between social cognitive and social motivational accounts of autism. Again we do not wish to frame cognitive, affective, and motor accounts of autism as mutually exclusive explanations; indeed, clinical, and basic science increasingly suggest that syndromes encompassing cognitive, affective, and motor coordination may be the rule rather than the exception (Gillberg, 2010).

This set of results also offers the possibility that in certain individuals with autism and oral motor impairment, expressive communication might be attained via gross and/or fine motor skills that can be somewhat more intact and may be more immediately or readily trainable relative to the level of oral motor skills. Such training of gross and fine motor skills prerequisite to communication may proceed via novel methods in traditional therapeutic settings (Chen et al., 2012) or via computer-assisted skills development as a tool for the therapist (Belmonte et al., 2013). There remains of course the potential that fine motor impairments could impede use of alternative and augmentative communication devices, because open-loop motor control which is unintegrated with sensory feedback (Haswell et al., 2009) leads to errors in pointing with a finger or hand to select amongst multiple response options. However, our current results do suggest that manual motor skills may be at least a more practical route to communication in these individuals than is spoken language. Most of all, these results highlighting autism's clinical heterogeneity in terms of motor function and ability to speak ought to prompt clinical and basic researchers and therapists to eschew a one-size-fits-all approach to autism: both therapeutic intervention and basic science must take note of such variability within the phenotype, and of the maxim that “If you've seen one person with autism, you've seen *one* person with autism.”

### Conflict of interest statement

The authors declare that the research was conducted in the absence of any commercial or financial relationships that could be construed as a potential conflict of interest.

## References

[B1] Aluri U. (2005). Oro-Motor Kit. Bangalore: The Com DEALL Trust.

[B2] Amato J. J. Slavin D. (1998). A preliminary investigation of oromotor function in young verbal and nonverbal children with autism. Infant-Toddler Intervent. Transdiscip. J. 8, 175–184

[B3] Archana G. (2008). A Manual from Communicaid: Assessment of Oro Motor Skills in Toddlers. Bangalore: The Com DEALL Trust.

[B4] Belmonte M. K. Cook E. H. Jr. Anderson G. M. Rubenstein J. L. R. Greenough W. T. Beckel-Mitchener A. (2004). Autism as a disorder of neural information processing: directions for research and targets for therapy. Mol. Psychiatry 9, 646–663 Available online at: http://www.cureautismnow.org/conferences/summitmeetings/ 10.1038/sj.mp.4001499 15037868

[B5] Belmonte M. K. Dhariwal M. Saxena-Chandhok T. Karanth P. (2013). Design of a touch-screen computer application to develop foundational motor communicative skills. Abstract presented at the International Meeting for Autism Research (San Sebastián).

[B6] Bishop D. V. (2002). Motor immaturity and specific speech and language impairment: evidence for a common genetic basis. Am. J. Med. Genet. 114, 56–63 10.1002/ajmg.1630 11840507

[B7] Chen G. M. Yoder K. J. Ganzel B. L. Goodwin M. S. Belmonte M. K. (2012). Harnessing repetitive behaviours to engage attention and learning in a novel therapy for autism: an exploratory analysis. Front. Psychol. 3:12 10.3389/fpsyg.2012.00012 22355292 PMC3280620

[B8] Chevallier C. Kohls G. Troiani V. Brodkin E. S. Schultz R. T. (2012). The social motivation theory of autism. Trends Cogn. Sci. 16, 231–239 10.1016/j.tics.2012.02.007 22425667 PMC3329932

[B9] Durkin M. (2013). The epidemiology of autism spectrum disorder: toward a more inclusive world. Keynote presented at the International Meeting for Autism Research (San Sebastián).

[B10] Flapper B. C. Schoemaker M. M. (2013). Developmental Coordination Disorder in children with specific language impairment: co-morbidity and impact on quality of life. Res. Dev. Disabil. 34, 756–763 10.1016/j.ridd.2012.10.014 23220052

[B11] Frith U. (1997). The neurocognitive basis of autism. Trends Cogn. Sci. 1, 73–77 10.1016/S1364-6613(97)01010-3 21223867

[B12] Gernsbacher M. A. Sauer E. A. Geye H. M. Schweigert E. K. Goldsmith H. H. (2007). Infant and toddler oral- and manual-motor skills predict later speech fluency in autism. J. Child Psychol. Psychiatry 49, 43–50 10.1111/j.1469-7610.2007.01820.x 17979963 PMC4123528

[B13] Gillberg C. (2010). The ESSENCE in child psychiatry: early symptomatic syndromes eliciting neurodevelopmental clinical examinations. Res. Dev. Disabil. 31, 1543–1551 10.1016/j.ridd.2010.06.002 20634041

[B14] Greenspan S. I. (2001). The affect diathesis hypothesis: the role of emotions in the core deficit in autism and the development of intelligence and social skills. J. Dev. Learn. Disord. 5, 1–45

[B15] Haswell C. C. Izawa J. Dowell L. R. Mostofsky S. H. Shadmehr R. (2009). Representation of internal models of action in the human brain. Nat. Neurosci. 12, 970–972 10.1038/nn.2356 19578379 PMC2740616

[B16] Hill E. L. (2001). Non-specific nature of specific language impairment: a review of the literature with regard to concomitant motor impairments. Int. J. Lang. Commun. Disord. 36, 149–171 10.1080/13682820010019874 11344592

[B17] Hill E. L. (2004). Executive dysfunction in autism. Trends Cogn. Sci. 8, 26–32 10.1016/j.tics.2003.11.003 14697400

[B18] Karanth P. (2007). Communication DEALL Developmental Checklists. Bangalore: The Com DEALL Trust.

[B19] Karanth P. (2010). Communication DEALL–The Program. Bangalore: The Com DEALL Trust.

[B20] Karanth P. Shaista S. Srikanth N. (2010). Efficacy of Communication DEALL—An indigenous early intervention program for children with autism spectrum disorders. Ind. J. Paediatr. 77, 957–962 10.1007/s12098-010-0144-8 20821283

[B21] Leary M. R. Hill D. A. (1996). Moving on: autism and movement disturbance. Ment. Retard. 34, 39–53 8822025

[B22] Marton K. (2009). Imitation of body postures and hand movements in children with specific language impairment. J. Exp. Child Psychol. 102, 1–13 10.1016/j.jecp.2008.07.007 18823904 PMC2584158

[B23] Noterdaeme M. Mildenberger K. Minow F. Amorosa H. (2002). Evaluation of neuromotor deficits in children with autism and children with a specific speech and language disorder *Eur.* Child Adolesc. Psychiatry 11, 219–225 10.1007/s00787-002-0285-z 12469239

[B24] Rechetnikov R. P. Maitra K. (2009). Motor impairments in children associated with impairments of speech or language: a meta-analytic review of research literature. Am. J. Occup. Ther. 63, 255–263 10.5014/ajot.63.3.255 19522134

[B25] Tesink C. M. Buitelaar J. K. Petersson K. M. van der Gaag R. J. Kan C. C. Tendolkar I. (2009). Neural correlates of pragmatic language comprehension in autism spectrum disorders. Brain 132, 1941–1952 10.1093/brain/awp103 19423680

[B26] Thurm A. Lord C. Lee L. Newschaffer C. (2007). Predictors of language acquisition in preschool children with autism spectrum disorders. J. Autism Dev. Disord. 37, 1721–1734 10.1007/s10803-006-0300-1 17180717

[B27] Wang M. V. Lekhal R. Aarø L. E. Schjølberg S. (2013). Co-occurring development of early childhood communication and motor skills: results from a population-based longitudinal study. Child. Care Health Dev. [Epub ahead of print] 10.1111/cch.12003 22970997

[B28] World Health Organization. (1993). The ICD-10 Classification of Mental and Behavioural Disorders: Diagnostic Criteria for Research. Geneva: World Health Organization.

[B29] Zelaznik H. N. Goffman L. (2010). Generalized motor abilities and timing behavior in children with specific language impairment. J. Speech Lang. Hear. Res. 53, 383–393 10.1044/1092-4388(2009/08-0204) 20360463 PMC3657549

